# Exploring the Correlation Between Fibrosis Biomarkers and Clinical Disease Severity in PLN p.Arg14del Patients

**DOI:** 10.3389/fcvm.2021.802998

**Published:** 2022-01-13

**Authors:** Stephanie M. van der Voorn, Mimount Bourfiss, Anneline S. J. M. te Riele, Karim Taha, Marc A. Vos, Remco de Brouwer, Tom E. Verstraelen, Rudolf A. de Boer, Carol Ann Remme, Toon A. B. van Veen

**Affiliations:** ^1^Division Heart and Lungs, Department of Medical Physiology, University Medical Center Utrecht, Utrecht, Netherlands; ^2^Division Heart and Lungs, Department of Cardiology, University Medical Center Utrecht, Utrecht, Netherlands; ^3^Department of Cardiology, University Medical Center Groningen, University of Groningen, Groningen, Netherlands; ^4^Heart Center, Department of Cardiology, Amsterdam University Medical Center, Location Academic Medical Center, Amsterdam, Netherlands; ^5^Department of Clinical and Experimental Cardiology, Heart Centre, Amsterdam Univeristy Medical Center, Location Academic Medical Center, University of Amsterdam, Amsterdam, Netherlands

**Keywords:** biomarkers, fibrosis, phospholamban (PLN), cardiomyopathy, collagen

## Abstract

**Background:** Pathogenic variants in *phospholamban* (*PLN*, like p. Arg14del), are found in patients diagnosed with arrhythmogenic (ACM) and dilated cardiomyopathy (DCM). Fibrosis formation in the heart is one of the hallmarks in *PLN* p.Arg14del carriers. During collagen synthesis and breakdown, propeptides are released into the circulation, such as procollagen type I carboxy-terminal propeptide (PICP) and C-terminal telopeptide collagen type I (ICTP).

**Aim:** To investigate if PICP/ICTP levels in blood are correlative biomarkers for clinical disease severity and outcome in *PLN* p.Arg14del variant carriers.

**Methods:** Serum and EDTA blood samples were collected from 72 *PLN* p.Arg14del carriers (age 50.5 years, 63% female) diagnosed with ACM (*n* = 12), DCM (*n* = 14), and preclinical variant carriers (*n* = 46). PICP levels were measured with an enzyme-linked immune sorbent assay and ICTP with a radio immuno-assay. Increased PICP/ICTP ratios suggest a higher collagen deposition. Clinical data including electrocardiographic, and imaging results were adjudicated from medical records.

**Results:** No correlation between PICP/ICTP ratios and late gadolinium enhancement (LGE) was found. Moderate correlations were found between the PICP/ICTP ratio and end-diastolic/systolic volume (both *r*_s_ = 0.40, *n* = 23, *p* = 0.06). PICP/ICTP ratio was significantly higher in patients with T wave inversion (TWI), especially in leads V4–V6, II, III, and aVF (*p* < 0.022) and in patients with premature ventricular contractions (PVCs) during an exercise tolerance test (*p* = 0.007).

**Conclusion:** High PICP/ICTP ratios correlated with clinical parameters, such as TWI and PVCs. Given the limited size and heterogeneity of the patient group, additional studies are required to substantiate the incremental prognostic value of these fibrosis biomarkers in *PLN* p.Arg14del patients.

## Introduction

Pathogenic variants in multiple genes encoding proteins that are crucially important for a proper functioning of cardiomyocytes predispose to, or are directly causative for several forms of cardiomyopathy. Phospholamban (PLN) is such a key protein and is involved in calcium (Ca^2+^) handling in cardiomyocytes. It is located at the sarcoplasmic reticulum (SR), where it regulates the function of sarco/endoplasmic reticulum Ca^2+^-ATPase2a (SERCA2a). Dephosphorylated PLN tightly binds to SERCA2a and thereby inhibits SR Ca^2+^ influx. On the other hand, phosphorylation of PLN relieves the inhibitory binding of PLN to SERCA2a and facilitates Ca^2+^ influx into the SR (1–3). Therefore, PLN plays a crucial role in contractility of the heart. There are several pathogenic variants known in the *PLN* gene that might cause cardiomyopathy which can ultimately lead to heart failure (HF) and (potentially) lethal ventricular arrhythmias (VA). One of such variants is a Dutch founder variant, a heterozygous deletion of arginine at position 14 (p.Arg14del). This variant is found both in patients diagnosed with arrhythmogenic (ACM) as well as dilated cardiomyopathy (DCM) (1). Phenotypically, these patients typically show low QRS voltages and T wave inversions (TWI) on electrocardiogram (ECG) recordings in the precordial leads, HF, VA, and myocardial fibrosis (1–4).

Fibrosis formation is one of the early hallmarks of disease in patients with a p.Arg14del variant (3). Progressive fibrosis formation appears especially in the epicardial left ventricular (LV) free wall (5). Pathological fibrosis formation is a reaction to replace apoptotic cardiomyocytes or excessive production of extracellular matrix (ECM) components, like collagens. The ECM, or interstitium, consists of interstitial fluid and several proteins (e.g., lamins, dystrophin) most of them being produced by fibroblasts, such as proteoglycans and collagen, the latter being the main part of the cardiac ECM (6). The most abundant types of collagen in the heart are collagen type I (85%) and collagen type III (11%) (7, 8). There is continuous turnover of collagen synthesis and breakdown as the half-life of collagen in the human heart is 90–120 days (8). In a healthy heart, collagen forms a network that provides structure to the cardiac muscle and strength during contraction (9). Under pathological conditions such as in *PLN* cardiomyopathy, collagen production is increased, which results in excessive fibrosis formation in the heart. In this setting, fibrosis may act as a substrate for VA (7), but it also disturbs contractile performance by affecting both contraction and relaxation. Collagen is synthesized by cardiac fibroblasts as pre-procollagen. In the ECM, procollagen is cleaved to collagen, and thereby the amino (PINP and PIIINP)- and carboxy (CICP, CIIICP) pro-peptides are released into the circulation. Degradation of collagen occurs with the help of metalloproteinases (MMPs), where MMPs cleave collagen fibers into fragments for further degradation. In the myocardium, different MMPs are expressed. MMP-1, MMP-8, and MMP-13, also known as collagenases, are highly specific for collagen types I and III and cleave the collagen into 2 fragments. These fragments will denature at physiological conditions. The denatured collagen fragments can be further digested by MMPs known as gelatinases, particularly MMP-2 and MMP-9 (10, 11). One such a breakdown fragment, C-terminal telopeptide collagen type I (ICTP), is also released into the circulation, similarly to propeptides during synthesis of collagen (8).

In the clinical setting, cardiac fibrosis is detectable *via* late gadolinium enhancement on cardiac magnetic resonance imaging (LGE-CMR), CMR-based T1 mapping or in tissue biopsies. A drawback of LGE-CMR is the inability to detect small patches and diffuse fibrosis, including the more subtle types of fibrosis where collagen heterogeneously intermingles with cardiomyocytes and is most often responsible for arrhythmogenesis (7, 9). T1 mapping is a relatively new approach that enables detection of subtle and diffuse fibrosis in high resolution, although this technique currently is not yet standardized and not routinely available (3, 9). Moreover, tissue biopsies are not frequently used due to their highly invasive character. Given these shortcomings, the field would benefit from new additional approaches to detect fibrosis that are fast, non-invasive, and cheap. For that purpose, assessment of biomarkers that reflect on total collagen turnover in relation to clinical outcome are of interest (12–16). When successful, such an approach facilitates frequent analysis which will support early detection of disease onset and disease progression in carriers (including family members), and potentially also identification of patients at risk for severe clinical outcomes [such as ventricular tachycardia (VT) and sudden cardiac death (SCD)]. In this study, we measured procollagen type I carboxy-terminal pro-peptide (PICP, as a marker of collagen synthesis) and ICTP (as a marker of collagen breakdown) in serum obtained from *PLN* p.Arg14del patients. The ratio between PICP/ICTP levels is proposed to reflect the balance between collagen synthesis and breakdown (17, 18). As such, we hypothesized that higher PICP/ICTP levels correlate to increased myocardial fibrosis formation in hearts of these patients, and in turn are related to clinical outcomes. Therefore, the aim of this study was to investigate the correlation of PICP/ICTP levels with clinical and outcome parameters in *PLN* variant carriers.

## Materials and Methods

### Study Population and Diagnosis

Serum and EDTA blood samples were obtained from 72 *PLN* p.Arg14del carriers, and were all collected on the same day during an event day for *PLN* p.Arg14del carriers. These were either carriers with an ACM or DCM diagnosis, or preclinical pathogenic variant carriers not fulfilling diagnostic criteria for a cardiomyopathy. The latter group consisted of patients that were either preclinical/asymptomatic and some with an intermediate phenotype. Diagnosis of ACM followed the 2010 Task Force Criteria score (TFC), where a TFC score ≥4 led to a definite ACM diagnosis (19). Diagnosis of DCM followed criteria, which included left ventricular dilatation (end-diastolic diameter (LVEDD) >112% of predicted for age and body surface area (BSA), or LV end-diastolic volume >2SD from reference value) and a reduced LV function (ejection fraction (LVEF) <45% or fractional shortening (FS) <25%) (20). Patients with a history of heart transplantation were excluded from this study. All subjects provided written informed consent. The study was conducted according to the Declaration of Helsinki. Blood samples were stored (AUMC-Durrer center Biobank number DC17-006) at −80°C until use.

### Clinical Data Collection

Clinical data were extracted from the ACM and PLN Registry as hosted in REDCap. These registries collect clinical data including demographics, symptoms, medication use, family history and genetic analysis (21).

Clinical parameters were categorized into functional/structural and electrical parameters. Functional/structural parameters included LGE-CMR, EF, end-diastolic and end-systolic volume (EDV, ESV, respectively). Dysfunction and dilatation parameters were qualitative assessments of ventricular function and diameter. For example, dilatation of the right ventricle (RV) was based on the RV diameter compared to the LV.

For electrical parameters QRS duration, TWI and low QRS voltages were assessed *via* ECG. A T wave was considered inverted if the voltage shifted ≥0.1 mV below baseline (21). A low QRS voltage was defined as a QRS peak-to-peak amplitude in leads I, II and III <0.5 mV or amplitude in all precordial leads <1.0 mV (22). Terminal activation duration (TAD) was defined as the longest value in V1–V3 from the nadir of the S wave to the end of all depolarization deflections in absence of a complete right bundle branch block, and was considered prolonged if ≥55 ms. During an exercise tolerance test the presence of premature ventricular contractions (PVCs) was assessed. Non-sustained VT was defined as three or more consecutive PVCs with a rate >100/min, lasting <30 s recorded on a Holter. For PVC amount in 24 h, all PVCs were counted except for VTs recorded on a Holter (22). Lastly, HF was defined as NYHA class II or higher, or ACC/AHA Stage C or higher (21).

Timepoint of serum collection was considered as “baseline” (T = 0) in this study. Clinical information around this date (with a time window of maximally 2 years prior to or following serum collection) were extracted from REDCap. To validate this chosen time window of 2 years, we did a sensitivity analysis including only patients who underwent clinical testing within a maximum of 5.5 weeks around blood sampling to get a closer picture of the reflection of PICP/ICTP levels and their relationship to clinical outcome (*n* = 12). Only a limited number of patients could be included into this sub-analysis, however, their PICP/ICTP levels were in line with the obtained overall picture (time window +/- 2 years), as depicted in red dots in Supplementary Figures 1A–D.

### Measurements of PICP and ICTP

PICP levels were measured using a commercially available enzyme-linked immune sorbent assay (ELISA) (MicroVue Bone, CICP, Cat #8005) according to the manufacturer's protocol. In short, diluted EDTA serum was administrated to murine monoclonal anti-CICP antibody coated strips. After incubation with rabbit anti-CICP antibodies, enzyme conjugate and substrate solution were added to the wells in different steps. After administrated stop solution to the wells, optical density was measured at 405 nm using the Bio-plex® 200 system (Bio-Rad, Hercules, CA).

ICTP levels were measured using a commercially available radioimmunoassay (RIA) (UniQ®, Orion Diagnostica, Cat #68601). Serum was incubated with Tracer, Antiserum and Procollagen Separation Reagent to test tubes. After centrifugation and decantation of the supernatant, tubes were counted using a gamma counter (Wizard^2®^, PerkinElmer). PICP and ICTP levels are shown as ng/mL.

### Statistics

Biomarker data were not normally distributed. Therefore, when continuous variables between two groups were compared, Mann-Whitney *U*-test was performed. For testing of multiple groups, Kruskal Wallis test with adjusted *p*-values by Bonferroni correction were used. Correlation between variables were assessed using Spearman's correlation coefficient. Data were considered significant if *p* < 0.05, correlations were considered weak between 0.10 and 0.40, moderate between 0.40 and 0.60, strong between 0.60 and 0.80 and very strong between 0.80 and 1.00. Data are shown as median [interquartile range]. Statistical analysis was performed using PRISM 9.0 (GraphPad Software, La Jolla, CA, USA, RRID:SCR_002798).

## Results

### Patient Characteristics

Patient characteristics are summarized in Table 1. The median age of all *PLN* variant carriers was 50.5 years [37–59 years], and 45/72 (63%) were female. In total, 12/72 (17%) patients were diagnosed with ACM according the 2010 TFC score [median TFC score 5 (4–7)], while 14/72 (19%) patients were diagnosed with DCM and 46/72 (64%) were preclinical variant carriers without phenotypic expression of cardiomyopathy (yet). No significant differences in age and sex were found between the three groups. The proband status (first affected family member) was significantly different between these subgroups (*p* = 0.01). Also, medication use such as betablockers and ACE-inhibitors significantly differed between the subgroups (*p* < 0.03). Similarly, ICD implantation varied between patients with ACM, patients with DCM, and preclinical variant carriers (*p* < 0.003).

**Table 1:** Patient characteristics of included *PLN* variant carriers.

	**All *PLN***	**ACM diagnosed**	**DCM diagnosed**	**Preclinical variant carrier**	**Adjusted**
	**(*n* = 72)**	**(*n* = 12)**	**(*n* = 14)**	**(*n* = 46)**	***p*-value**
**Demographics**					
Age (years)	50.50 [37–59]	53 [48.75–56]	56 [43.50–61.25]	46 [37–58.75]	>0.999
Female	45/72 (63)	7/12 (58)	7/14 (50)	31/46 (67)	>0.999
Proband status	21/72 (29)	6/12 (50)	9/14 (64)	5/46 (11)	0.01^**^
TFC score	2 [0–3]	5 [4–7]	2 [1.25–3]	1 [0–2]	<0.003^**^
**Treatment**					
Betablockers	29/71 (41)	5/12 (42)	13/14 (93)	11/45 (24)	<0.003^**^
Antiarrhythmics	6/71 (8)	2/12 (17)	3/14 (21)	1/45 (2)	>0.999
Diuretics	16/71 (23)	4/12 (33)	6/14 (43)	6/45 (13)	>0.999
ACE-inhibitors	26/71 (37)	5/12 (42)	11/14 (79)	10/45 (22)	0.024^*^
ICD implantation	27/72 (38)	7/12 (58)	11/14 (79)	9/46 (20)	<0.003^**^
**Imaging/MRI**	1.03 years				
LGE LV	9/23 (39)	2/4 (50)	2/2 (100)	5/17 (29)	>0.999
LVEF (%)	54 [51.55–57.75]	54 [53–55]	39.50 [38.75–40.25]	55 [52–58]	>0.999
LV EDV (mL)	161.29 [152–189.40]	170 [157–175]	307.50 [272.75–342.25]	158 [152–178.80]	>0.999
LV ESV (mL)	74.14 [66–90.50]	75 [69.75–80]	185.50 [162.25–208.75]	73 [66–76]	>0.999
LGE RV	1/22 (5)	0/4 (0)	0/1 (0)	1/17 (6)	>0.999
RVEF (%)	53 [47–56]	55 [49.50–56.50]	-	54 [50.50–57.50]	>0.999
RV EDV (mL)	167 [123–193.70]	181 [151–191.50]	-	154.50 [121.50–181]	>0.999
RV ESV (mL)	81 [56.25–109]	81 [66–97]	-	76 [55.82–100.50]	>0.999
**Echocardiogram**	0.64 years				
LV dysfunction	24/61 (39)	5/10 (50)	11/13 (85)	8/38 (21)	0.007^**^
LV dilatation	11/59 (19)	4/10 (40)	7/13 (54)	0/36 (0)	<0.003^**^
RV dysfunction	11/61 (18)	7/10 (70)	3/13 (23)	1/38 (3)	<0.003^**^
RV dilatation	7/57 (12)	4/8 (50)	0/11 (0)	3/38 (8)	0.065
**ECG-monitoring**	0.49 years				
QRS duration (ms)	92 [80.50–112.25]	97.50 [93–102.50]	98 [84.50–118.25]	89 [78–108.50]	>0.999
T wave inversion	36/56 (64)	7/9 (78)	10/14 (71)	19/33 (58)	>0.999
Number of leads with TWI out of 9	2 [0–5]	3 [1–5]	3 [0.5–5]	1 [0–2]	>0.999
Low QRS voltage	22/63 (35)	5/11 (45)	10/14 (71)	7/38 (18)	0.058
TAD duration ≥55 ms	6/50 (12)	3/8 (38)	1/13 (8)	2/29 (7)	>0.999
**Exercise tolerance test**	0.60 years				
PVCs	30/43 (70)	5/7 (71)	7/7 (100)	18/29 (62)	>0.999
**Holter**					
Non-sustained VT	5/33 (15)	2/7 (29)	2/2 (100)	1/24 (4)	0.03^*^
PVC amount in 24 h	387 [7.5–682.5]	534.5 [412.25–3,190.25]	5,850.5 [5,172.75–6,528.25]	163 [–491]	>0.999
**Outcomes**					
Heart failure	11/70 (14)	2/12 (17)	8/14 (57)	0/44 (0)	<0.003^**^
VT/VF event or appropriate ICD shock	13/62 (21)	3/9 (33)	6/11 (55)	4/42 (10)	0.112

*Data is depicted as median [interquartile range], n (%), or n/N (%). PLN, phospholamban; ACM, arrhythmogenic cardiomyopathy; DCM, dilated cardiomyopathy; TFC, 2010 Task Force Criteria; ACE-inhibitors, angiotensin-converted enzyme inhibitors; ICD, implantable cardioverter-defribrillator; LGE, late gadolinium enhancement; LVEF, left ventricular ejection fraction; EDV, end-diastolic volume; ESV; end-systolic volume; RVEF, right ventricular ejection fraction; TAD, terminal activation duration; PVCs, premature ventricular contractions; VT/VF, ventricular tachycardia/ventricular fibrillation. Kruskal wallis test is performed, Bonferroni correction is applied to correct p-value*,

** *p < 0.01*,

* *p < 0.05*.

No significant difference in EF, EDV, or ESV of the ventricles was found between the three subgroups. However, the median of LVEF in DCM group was 40%, for ACM diagnosed 54% and preclinical pathogenic variant carriers 55%. The median LV EDV for DCM patients was 308 mL, for ACM 170 mL and for preclinical variant carriers 158 mL. For LV ESV, the median for the DCM diagnosed patients was 186 mL, ACM diagnosed 75 mL and for preclinical pathnogenic variant carriers 73 mL. Furthermore, nine out of 23 patients showed LGE in the LV, while one out of 22 patients showed LGE in the RV. As expected, in ACM classified *PLN* patients the RV was significantly more dysfunctional, while in DCM diagnosed *PLN* patients primarily the LV was affected, as ventricular dysfunction was assessed *via* echocardiogram analysis.

No difference in QRS duration, TWI, number of leads with TWI and the presence of a TAD of ≥55 ms or low QRS voltage were found with ECG-recordings between the subgroups. The presence of PVCs at baseline and during an exercise tolerance test did not differ between the subgroups. Detection of non-sustained VTs with a Holter-recording showed significant differences between ACM or DCM diagnosed and preclinical variant carriers (*p* = 0.03). The median amount of PVCs recorded in 24 h on a holter for DCM patients was 5,850.5 [5,172.75–6,528.25], for ACM patients 534.5 [412.25–3,190.25] and for preclinical variant carriers 163 [6–491] Lastly, a significant difference between the incidence of HF (*p* < 0.003) was found between ACM, DCM patients, and preclinical variant carriers.

### Fibrosis Biomarkers

The median level of PICP for all 72 *PLN* p.Arg14del patients was 120.18 [98.32–146.98] ng/mL, for ICTP 3.14 [2.42–3.81] ng/mL and a PICP/ICTP ratio of 42.40 [29.70–51.63]. There was no influence of sex or medication use on the PICP/ICTP levels (Supplementary Figure 2).

Furthermore, no differences were observed in PICP, ICTP, and PICP/ICTP levels within ACM (PICP; 112.55 [95.19–135.32] ng/mL, ICTP; 3.34 [2.68–4.76] ng/mL, and PICP/ICTP; 37.74 [23.54–45.37], *p* > 0.999), DCM (PICP; 133.44 [104.91–156.28] ng/mL, ICTP; 3.36 [2.38–3.87] ng/mL, and PICP/ICTP; 43.70 [27.90–51.88], *p* > 0.999) patients and preclinical pathogenic variant carriers [PICP; 120.18 (98.62–144.72) ng/mL, ICTP; 3.07 (2.43–3.54) ng/mL, and PICP/ICTP; 42.57 (31.53–54.12) *p* > 0.999], as summarized in Table 2.

**Table 2:** Fibrosis biomarkers concentrations in all *PLN* variant carriers (*n* = 72) or divided into ACM diagnosed (*n* = 12), DCM diagnosed (*n* = 14) or preclinical pathogenic variant carriers (*n* = 46).

	**All *PLN***	**ACM diagnosed**	**DCM diagnosed**	**Preclinical variant carrier**	**Adjusted**
	**(*n* = 72)**	**(*n* = 12)**	**(*n* = 14)**	**(*n* = 46)**	***p*-value**
PICP (ng/mL)	120.18 [98.32–146.98]	112.55 [95.19–135.32]	133.44 [104.91–156.28]	120.18 [98.62–144.72]	>0.999
ICTP (ng/mL)	3.14 [2.42–3.81]	3.34 [2.68–4.76]	3.36 [2.38–3.87]	3.07 [2.43–3.54]	>0.999
PICP/ICTP	42.40 [29.70–51.63]	37.74 [23.54–45.37]	43.70 [27.90–51.88]	42.57 [31.53–54.12]	>0.999

*Data is shown as median [interquartile range]. PLN, phospholamban; ACM, arrhythmogenic cardiomyopathy; DCM, dilated cardiomyopathy; PICP, procollagen type I carboxy-terminal pro-peptide; ICTP, C-terminal telopeptide collagen type I. Kruskal wallis test is performed. Bonferroni correction is applied to correct p-value*.

### Correlation of Fibrosis Biomarkers With Structural/Functional Parameters

**Figure 1:**
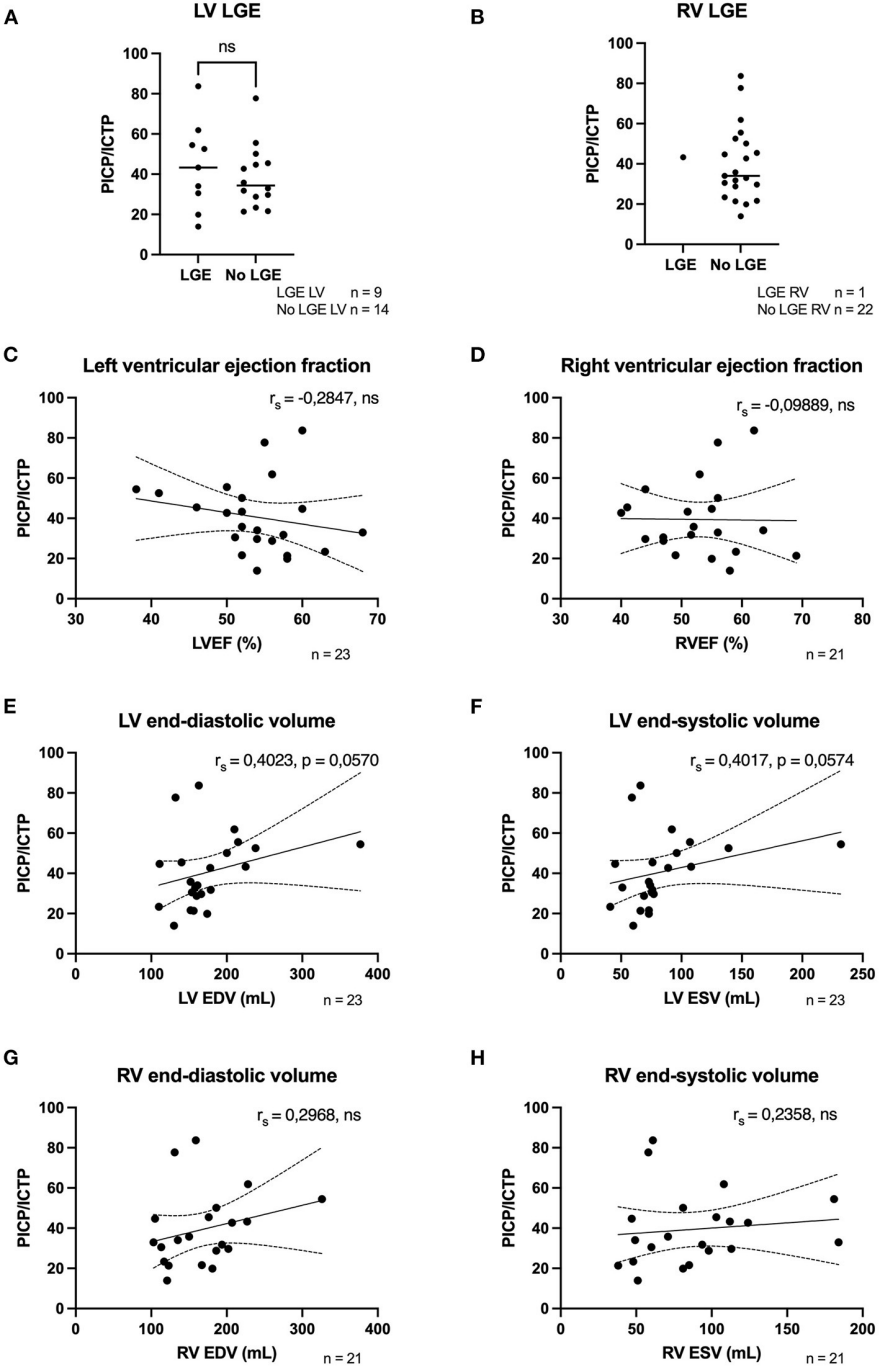
Fibrosis biomarkers correlated weakly to moderatly to structural/functional parameters. **(A)** PICP/ICTP ratio was not different in patients with LV LGE (*n* = 9) compared to patients who did not have LV LGE (*n* = 14). **(B)** Only one patient had LGE in the RV, therfore no comparison could be made. **(C)** A weak negative correlation was found with LVEF (*n* = 23). **(D)** No correlation was found with RVEF and fibrosis biomarkers (*n* = 21). **(E,F)** Moderate positive correlations were found with LV EDV and ESV (*n* = 23). **(G,H)** Weak positive correlations were found with RV EDV and ESV (*n* = 21). LGE, late gadolinium enhancement; LVEF, left ventricular ejection fraction; RVEF, right ventricular ejection fraction; EDV, end-diastolic volume; ESV, end-systolic volume; PICP, procollagen type I carboxy-terminal pro-peptide; ICTP, C-terminal telopeptide collagen type I; rs, Spearman's rho; ns, not significant.

LGE-CMR was performed in a subset of 23 patients included in our cohort. No differences between PICP/ICTP ratios were found between patients that showed LV fibrosis and the patients who did not; 43.30 [30.56–54.43] compared to 34.36 [29.01–45.27], *p* = 0.60 (Figure 1A). For the RV, only one preclinical variant carrier revealed significantly enhanced LGE (Figure 1B).

As for functional parameters, LVEF showed a weak, but not significant, inverted correlation (*r*_s_ = −0.28, *p* = 0.188) with PICP/ICTP ratio (Figure 1C). For RVEF, no correlation was found (*r*_s_ = −0.10, *p* = 0.67) with PICP/ICTP, Figure 1D.

For the morphologic parameters (Figures 1E,F), moderate positive correlations were found with LV EDV and LV ESV volume (both *r*_s_ = 0.40, *p* = 0.06). Weak, albeit not significant, positive correlations were found for RV EDV (*r*_s_ = 0.30, *p* = 0.297) and RV ESV (*r*_s_ = 0.24, *p* = 0.304) as shown in Figures 1G,H. To summarize, weak to moderate, but not significant, correlations were found with structural/functional parameters and fibrosis biomarkers.

### Correlation of Fibrosis Biomarkers With Electrical Parameters

**Figure 2:**
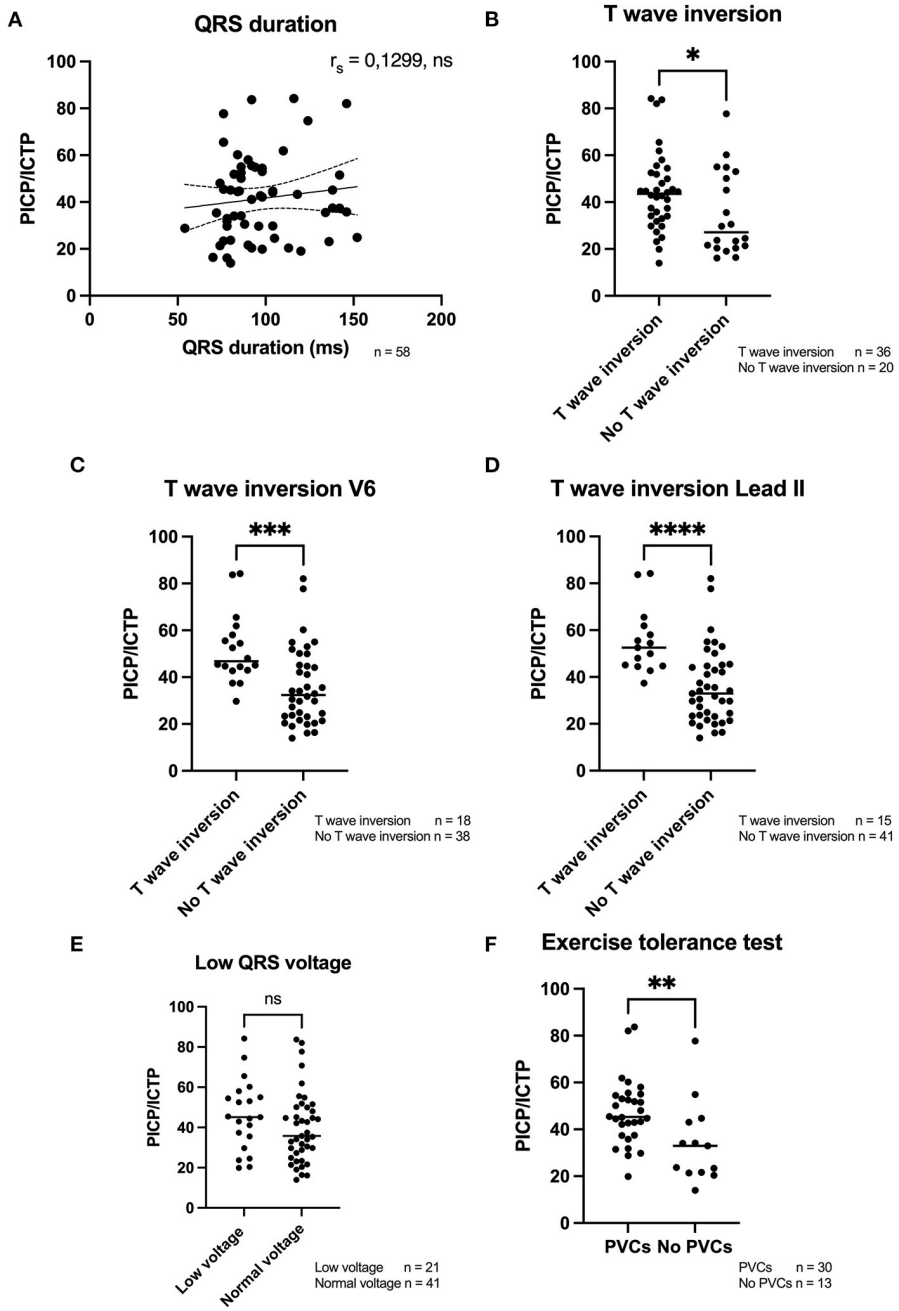
Fibrosis biomarkers in relation to electrical parameters. **(A)** A weak positive correlation was found with QRS duration (*n* = 58). **(B–D)** A significant higher PICP/ICTP ratio was found when a T wave inversion was present, overall, but especially when detected in lead V4–V6 and lead II, III, and aVF. **(E)** No difference was found in the ratio of fibrosis biomarkers and the presence or absence of low QRS voltages. **(F)** PICP/ICTP ratio was significantly higher when PVCs were detected during an exerice tolerance test. PVCs, premature ventricular contractions; PICP, procollagen type I carboxy-terminal pro-peptide; ICTP, C-terminal telopeptide collagen type I; rs, Spearman's rho. ****p < 0.0001, ***p < 0.001, **p < 0.01, *p < 0.05.

Previous research revealed an association between the proportion of myocardial fibrosis and QRS duration in a population of SCD patients (23). In our study, only a weak and not significant correlation between QRS duration and PICP/ICTP ratio was found (*r*_s_ = 0.13, *p* = 0.331), Figure 2A. On the other hand, the group of patients who presented with TWI had a significantly higher total collagen turnover [PICP/ICTP ratio 43.53 (33.77–52.02)] compared to the group of patients that did not have TWI [PICP/ICTP 27.11 (21.14–50.85), *p* = 0.044], Figure 2B. Moreover, when the different leads of the ECG (in particular leads V4–V6) were assessed, the presence of TWI was associated with a significantly higher collagen turnover [lead V4; 44.92 (41.52–56.67) compared to 34.07 (23.43–50.06), *p* = 0.022, lead V5; 45.12 (41.91–56.81) compared to 32.96 (23.09–45.46), *p* = 0.002, lead V6; 46.76 (43.33–57.43) compared to 32.36 (23.16–44.99), *p* = 0.001], as exemplified for lead V6 in Figure 2C. Also, significantly higher PICP/ICTP ratios were found when TWIs were present in lead II [52.54 (44.92–59.97) compared to 32.96 (23.34–44.69), *p* < 0.001], lead III [46.58 (36.16–55.29) compared to 34.84 (23.43–45.36), *p* = 0.022] and lead aVF [49.92 (44.73–55.57) compared to 34.10 (23.51–45.27), *p* = 0.004] were observed in *PLN* variant carriers, Figure 2D and summarized in Table 3.

**Table 3:** The PICP/ICTP ratios of patients who showed a T wave inversion compared to patients who did not show a T wave inversion in different leads of the ECG.

	**T wave inversion**	**No T wave inversion**	***p*-value**
Lead V4	44.92 [41.52–56.67]	34.07 [23.43–50.06]	0.022^*^
Lead V5	45.12 [41.91–56.81]	32.96 [23.09–45.46]	0.002^**^
Lead V6	46.76 [43.33–57.43]	32.36 [23.16–44.99]	0.001^***^
Lead II	52.54 [44.92–59.97]	32.96 [23.34–44.69]	<0.001^****^
Lead III	46.58 [36.16–55.29]	34.84 [23.43–45.36]	0.022^*^
Lead aVF	49.92 [44.73–55.57]	34.10 [23.51–45.27]	0.004^**^

*Data is depicted as median [interquartile range]. PICP, procollagen type I carboxy-terminal pro-peptide; ICTP, C-terminal telopeptide collagen type I; ECG, electrocardiogram. Mann-Whintey U-test is performed*.

**** *p < 0.0001*,

*** *p < 0.001*,

** *p < 0.01*,

* *p < 0.05*.

Another clinical manifestation in *PLN* p.Arg14del carriers is the presence of low QRS voltages (3). Our data did not identify a correlation between the presence of these low voltages and high collagen turnover, Figure 2E. However, PICP/ICTP ratios were significantly higher in patients who presented with PVCs during an exercise tolerance test [45.29 (38.62–54.09)] as compared to patients in which no PVCs occurred [32.96 (21.64–43.05), *p* = 0.007], as depicted in Figure 2F.

### Subpopulation Analysis of Electrical Parameters

**Figure 3:**
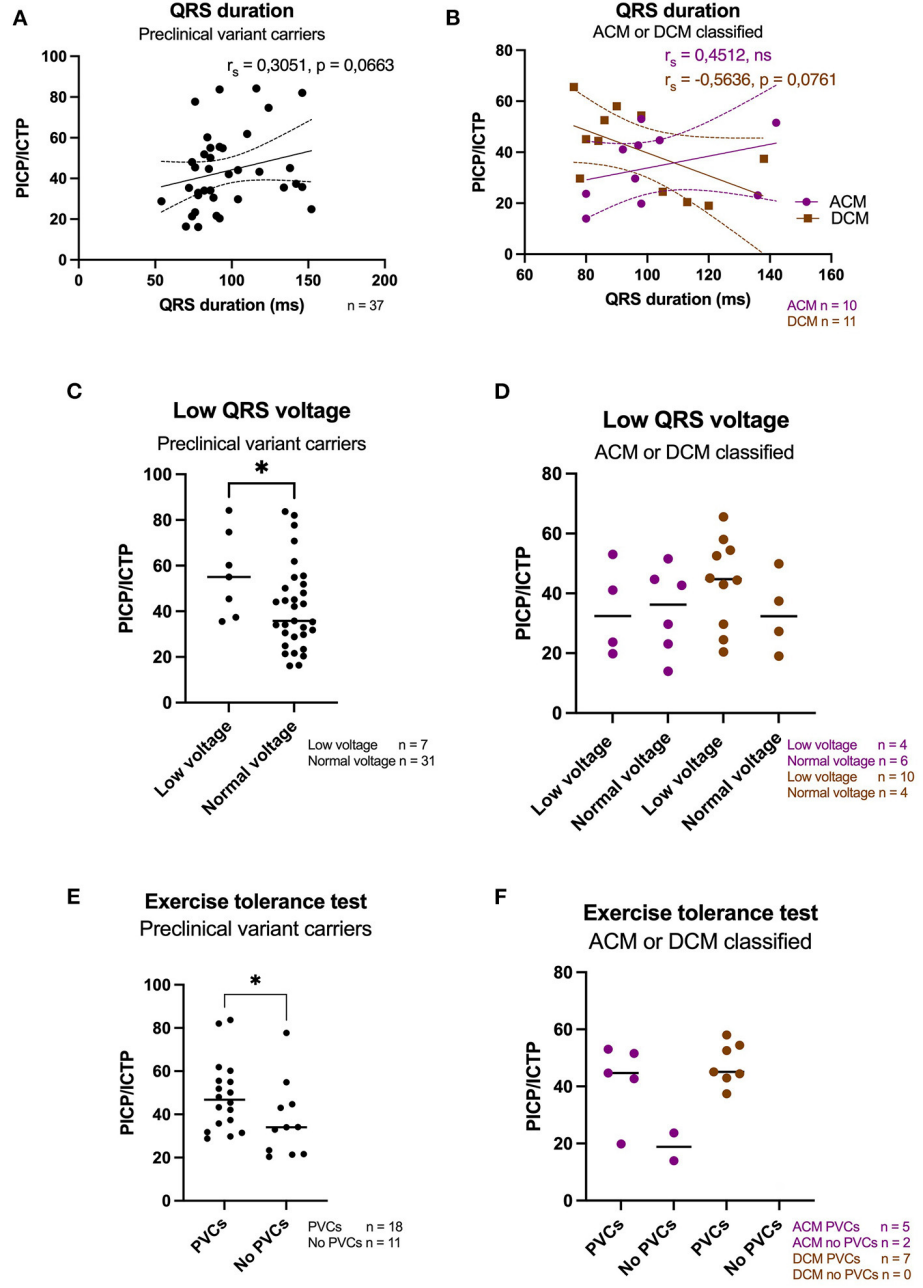
Subpoulation analysis of electrical parameters. **(A)** A weak positive correlation was found with QRS duration in preclinical pathogenic variant carriers (*n* = 37). **(B)** A moderate positive correlation was found with QRS duration in patients diagnosed with ACM (*n* = 10), while a moderate negative correlation was found with QRS duration in DCM diagnosed patients (*n* = 11). **(C)** A significant higher PICP/ICTP ratio was found in preclinical variant carriers that presented with low QRS voltages. **(D)** No significant difference was found in ACM or DCM diagnosed *PLN* patients with total collagen turnover and the presence of low QRS voltages. **(E)** A significant higher PICP/ICTP ratio was found when PVCs were detected during an exericse tolerance test in preclinical variant carriers. **(F)** No signficant difference was found in PICP/ICTP ratios and the detection of PVCs in ACM or DCM classified *PLN* patients. Of note, all DCM patients presented with PVCs during an exericse tolerance test (*n* = 7). ACM, arrhythmogenic cardiomyopathy; DCM, dilated cardiomyopathy; PVCs, premature ventricular contractions; PICP, procollagen type I carboxy-terminal pro-peptide; ICTP, C-terminal telopeptide collagen type I; rs, Spearmann's rho. *p < 0.05.

Since we included a heterogeneous study population, with some patients diagnosed with ACM or DCM, and preclinical variant carriers without a clinical diagnosis of a cardiomyopathy, a subpopulation analysis was performed. For preclincial variant carriers, a weak but not significant correlation between prolonged QRS duration and increased PICP/ICTP ratios (*r*_s_ = 0.31, *p* = 0.066) was found, Figure 3A. Patients diagnosed with an ACM phenotype showed a similar correlation as the preclinical variant carriers (*r*_s_ = 0.45, *p* = 0.192), whereas on the other hand DCM diagnosed *PLN* patients showed an inverted relationship with profibrotic biomarkers (*r*_s_ = −0.56, *p* = 0.076), Figure 3B. When the presence of low QRS voltages was compared among groups, we found a significant higher collagen turnover in preclinical variant carriers that showed low QRS voltages, but this discrimination was absent in the ACM and DCM diagnosed patients, Figures 3C,D. Similarly to the whole group analysis, preclinical *PLN* p.Arg14del variant carriers had a significantly higher total collagen turnover when they experienced PVCs during an exercise tolerance test, Figure 3E. In contrast, no significant difference was observed in the ACM or DCM diagnosed group. Of note, the small group of only 7 DCM patients all showed PVCs with high PICP/ICTP biomarker levels, as shown in Figure 3F.

## Discussion

To date, visualization of especially patchy and diffuse fibrosis is difficult with current clinical techniques, so an experimental approach using biomarkers might help to provide better insights into profibrotic remodeling of the heart under pathophysiological conditions. In this study, we found weak to moderate correlations of profibrotic biomarkers with QRS duration, EF, EDV, and ESV in both LV and RV in *PLN* p.Arg14del carriers. Of note, our study population was quite heterogeneous, as it included patients diagnosed with ACM or DCM, but the majority concerned pathogenic variant carriers that did not have a clinical diagnosis of cardiomyopathy (yet). Therefore, an additional subpopulation analysis was performed. This showed a weak but not significant correlation of prolonged QRS duration with profibrotic markers in the blood, but only for preclinical variant carriers. Additionally, this group of preclinical *PLN* p.Arg14del variant carriers that showed low QRS voltages and TWI had significant higher PICP/ICTP ratios compared to the group that did not have these ECG changes.

An excessive production of collagen hinders cardiac function by: (1) hampering electrical conduction and thereby increasing the risk for VA, (2) reducing contractile performance of the ventricles, and (3) increasing ventricular stiffness thereby impairing the filling and relaxation capacity of the ventricle (7, 24). Electrical parameters, such as prolonged QRS duration and the presence of low QRS voltages on the ECG are suggested to reflect myocardial fibrosis (4, 23, 25). Furthermore, TWI can indicate the presence of a repolarization defect whereas also abnormal depolarization can result in a TWI. In our study, both these low QRS voltages and TWI in lead V5, V6, II, and aVF were also found in *PLN* p.Arg14del variant carriers without an ACM or DCM diagnosis, and thereby might reflect an early electrical transformation in still relatively unaffected patients (3, 4). The observation that significantly increased PICP/ICTP ratios correlated to TWI especially in ECG leads V4–V6 might suggest that the LV is more affected in most *PLN* p.Arg14del patients. When we compared ventricular dysfunction in *PLN* p.Arg14del carriers diagnosed with ACM or DCM, as expected, the ACM patients showed predominantly RV dysfunction while DCM diagnosed patients showed more LV dysfunction (see Table 1). More proof of electrical instability in *PLN* p.Arg14del patients with a high total collagen turnover adhered to the presence of PVCs during an exercise tolerance test. PVCs are early depolarizations of the ventricle which are linked to increased risk of severe arrhythmias and SCD (26). Transgenic mice expressing the *PLN* p.Arg14del variant also showed accumulation of myocardial fibrosis and contractile dysfunction by reduced LVEF and increased EDV and ESV. The contractile impairment most likely results from inhibition of SERCA by the pathogenic *PLN* variant, which causes a distortion in Ca^2+^ cycling. Moreover, these *PLN* mutated mice showed lower QRS voltages and increased incidence of VA *ex vivo* (1).

Previous research compared PICP levels in blood to the extent of myocardial fibrosis in endocardial biopsies in HF patients. PICP levels were elevated in blood obtained from both the coronary artery and peripheral blood, which suggest that PICP levels measured in peripheral blood reflects collagen turnover in the heart. Although values obtained from coronary blood overall were higher than measured in peripheral blood, a direct correlation was found between the degree of endocardial fibrosis and the PICP levels both in coronary blood and peripheral blood. More importantly, a direct correlation existed between PICP levels measured in coronary and peripheral blood and collagen type I deposition in the heart (16). This suggests that PICP levels measured in peripheral blood (like we analyzed in our study) indeed reflects collagen turnover in the heart. Substantiating the findings in our study, elevated PICP levels were shown to be highly sensitive and specific for identification of severe myocardial fibrosis in a study of patients suffering from arterial hypertension (16). Similarly a study of *Aguiar* et al. identified a correlation of PICP/ICTP with LGE in patients with Fabry disease cardiomyopathy (27), but this correlation with LGE was absent in a cohort of overt hypertrophic cardiomyopathy (HCM) patients and HCM pathogenic variant carriers without left ventricular hypertrophy (17).

In this cohort of HCM patients, it was however shown that the PICP/ICTP ratio was increased in overt HCM patients, but not in pathogenic variant carriers, suggesting that in overt disease state, collagen synthesis exceeded collagen breakdown, while in preclinical variant carriers this increased collagen synthesis is stabilized by increased collagen breakdown (17). Another explanation, as formulated by *Lombardi* et al., suggested that collagen turnover is only robustly upregulated during a temporal progressive/hot phase of the disease (14). In Fabry disease cardiomyopathy it was shown that PICP/ICTP ratio was increased in patients with severe disease expression. Weak but significant correlations were found with fibrosis biomarkers and clinical outcome such as LV mass (28). Other studies also revealed weak, but significant, correlations between LVEF or left atrial (LA) diameter and PICP or ICTP levels in a population of elderly HF patients (13). Similarly, in hypertensive patients with chronic HF, weak but significant correlations were found with pulmonary capillary wedge pressure, a way to estimate filling pressure in these hypertensive patients (15). On the contrary, the study of *Querejeta* et al. did not find any significant correlations of PICP levels with clinical outcome in patients suffering from arterial hypertension, however this study population only consisted of 26 patients (16). In DCM patients, it was found that increased ICTP levels were associated with higher relative risk of advanced clinical disease progression and heart transplantation (12). In addition, it was found that increased ICTP levels correlated to a lower degree of cardiac event-free events (29).

In our study, we found weak to moderate correlations of PICP/ICTP levels to different parameters of clinical outcome. To our knowledge, this is the first time that these levels were measured in *PLN* p.Arg14del patients, and the degree in correlations fits with the correlation coefficients reported in the studies mentioned above. In a previous study performed in our group, the correlation of PICP/ICTP levels with myocardial fibrosis was investigated in which levels of fibrosis markers were measured at baseline, at 6 weeks and around 5 months after myocardial infarction (MI) in patients. We found that total collagen turnover was significantly increased after 6 weeks and 5 months. In addition, moderate correlations were found with increased PICP levels and scare size, as measured with LGE (manuscript under consideration). The stronger correlations in that particular study population most likely reflect the more severe degree of fibrosis formation upon MI.

## Limitations

Limitations of this study include the difference in timepoints between blood sampling and clinical assessment. Therefore, direct effects of analyzed PICP and ICTP levels cannot signal a temporal hot phase of the disease, but we assumed that the PICP and ICTP levels reflect a continuous progression of collagen deposition. Another limitation of the study is that we could not correct for other abnormalities potentially leading to collagen deposition, since collagen type I is not only produced in the heart but found in many other organs in the body. Therefore, it is possible that in some cases the PICP and ICTP levels do not exclusively reflect myocardial fibrosis formation. Finally, to strengthen the predictive character of PICP/ICTP ratio as biomarker, inclusion of more patients would have been preferred but this was unfortunately hampered by the fact that blood samples of patients with this rare pathogenic variant is limited. Therefore, additional preferable correlations made to fibrosis parameters such as to LGE appeared not to be possible. Although not considered as a real limitation, and in order to be clear, following the aim of this study to correlate the collagen biomarkers to clinical outcome we did not implement healthy control subjects. This is logical due to the fact that from this tentative additional group clinical records are obviously lacking.

## Conclusion

In conclusion, our data show that total collagen turnover correlates weakly to moderately with clinical parameters in *PLN* p.Arg14del patients. Contractile impairment of the LV also correlated weakly to moderately to total collagen turnover. In addition, we found a higher total collagen turnover in patients with PVCs and TWI, especially for leads V4–V6. However, to gain insights into the predictive value of PICP/ICTP ratio as a biomarker for clinical outcome, further investigation with a higher number of patients is required, especially in *PLN* variant carriers being diagnosed with either ACM or DCM.

## Data Availability Statement

The original contributions presented in the study are included in the article/Supplementary Material, further inquiries can be directed to the corresponding author/s.

## Ethics Statement

The studies involving human participants were reviewed and approved by AUMC-Durrer Center Biobank number DC17-006. The patients/participants provided their written informed consent to participate in this study.

## Author Contributions

SV, CR, and TABV contributed to study design, data collection, data analysis, and writing the manuscript. MB, AR, KT, RB, TEV, and RB contributed to the data collection and interpretation. All authors have read the manuscript and approved the final manuscript.

## Funding

This work was supported by a grant from the Netherlands Cardio Vascular Research Initiative (CVON): the Dutch Heart Foundation, Dutch Federation of University Medical Centers, the Netherlands Organization for Health Research and Development and the Royal Netherlands Academy of Sciences (CVON-eDETECT 2015-12, CVON-PREDICT2 2018-30, all authors). Further financial support was obtained from the Netherlands Heart Institute in conjunction with the PLN patient foundation (to TABV).

## Conflict of Interest

The authors declare that the research was conducted in the absence of any commercial or financial relationships that could be construed as a potential conflict of interest.

## Publisher's Note

All claims expressed in this article are solely those of the authors and do not necessarily represent those of their affiliated organizations, or those of the publisher, the editors and the reviewers. Any product that may be evaluated in this article, or claim that may be made by its manufacturer, is not guaranteed or endorsed by the publisher.
