# Supplementary material for: Exploring the Correlation Between Fibrosis Biomarkers and Clinical Disease Severity in PLN p.Arg14del Patients
Source: Front Cardiovasc Med. 2022 Jan 13;8:802998. doi: 10.3389/fcvm.2021.802998 (PMC8793805; doi:10.3389/fcvm.2021.802998)
Supplement: Supplementary file 1 [file Data_Sheet_1.docx]

Supplemental Figures

**S_Figure 1** Testing the time window of two years of clinicial data collection around blood sampling in *PLN* patients. Red dots represent patients that had clinical testing within 5.5 weeks around blood sampling. Black dots represents clinical data collection between 5.5 weeks and two years. These red dots are in line with the overall picture of +/- two years around blood sampling. PICP; procollagen type I carboxy-terminal pro-peptide, ICTP; C-terminal telopeptide collagen type I.

**S_Figure 2** The influence of sex and medication use on PICP/ICTP ratios. A) No difference in PICP/ICTP ratio was found between females and males. B) The use of betablockers did not alter the PICP/ICTP ratio in *PLN* patients. C) Antiarrhythmics did not changed the PICP/ICTP ratio. D) No difference in total collagen turnover in patients who used diuretics was found. E) The use of ACE-inhibitors did not alter the PICP/ICTP ratio. ACE-inhibitors; angiotensin-converted enzyme inhibitors PICP; procollagen type I carboxy-terminal pro-peptide, ICTP; C-terminal telopeptide collagen type I.
