# Supplementary figures and images for: Exploring the Correlation Between Fibrosis Biomarkers and Clinical Disease Severity in PLN p.Arg14del Patients
Source: Front Cardiovasc Med. 2022 Jan 13;8:802998. doi: 10.3389/fcvm.2021.802998 (PMC8793805; doi:10.3389/fcvm.2021.802998)

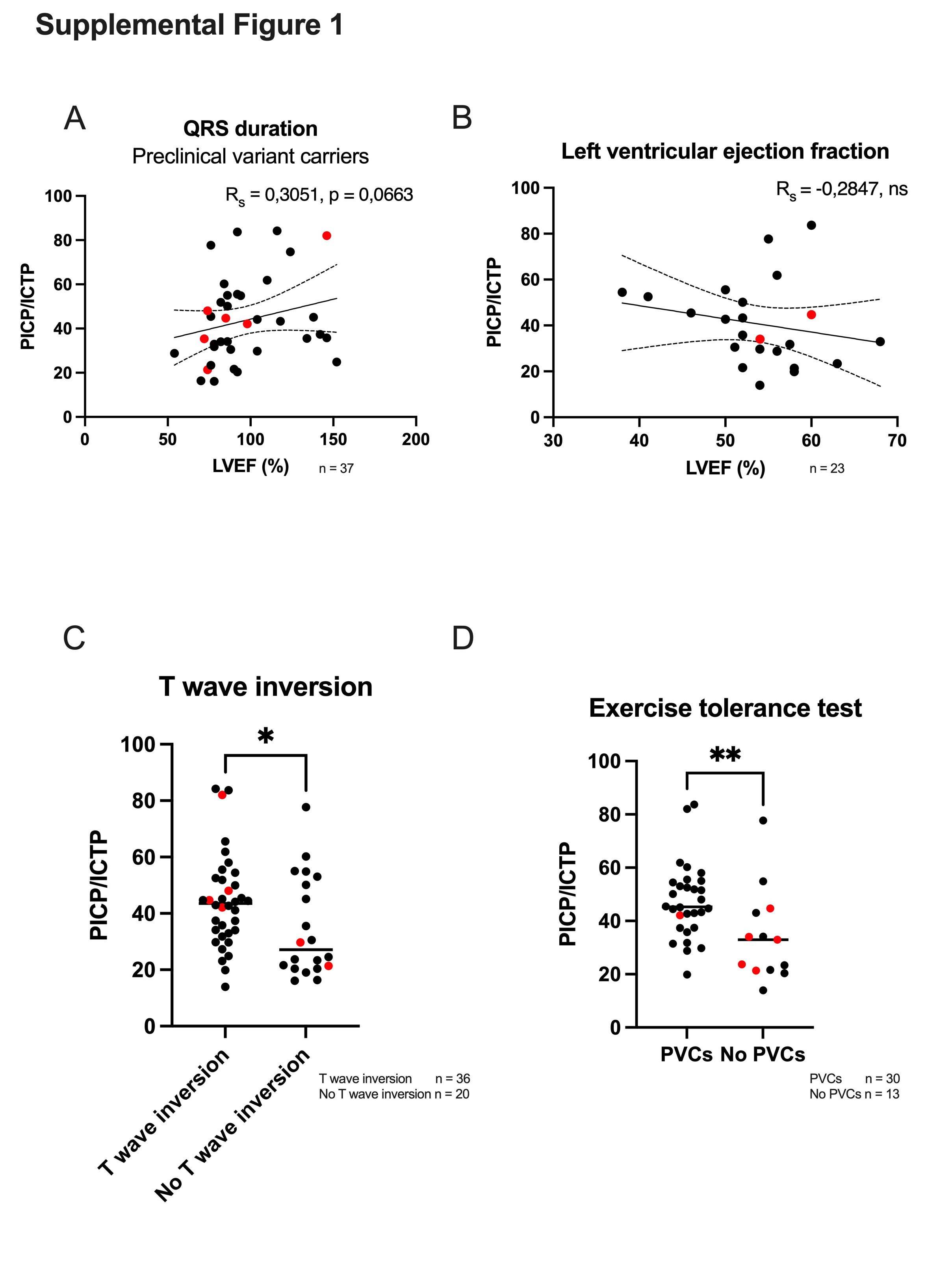

Supplement: Supplementary file 2 [file Image_1.JPEG]

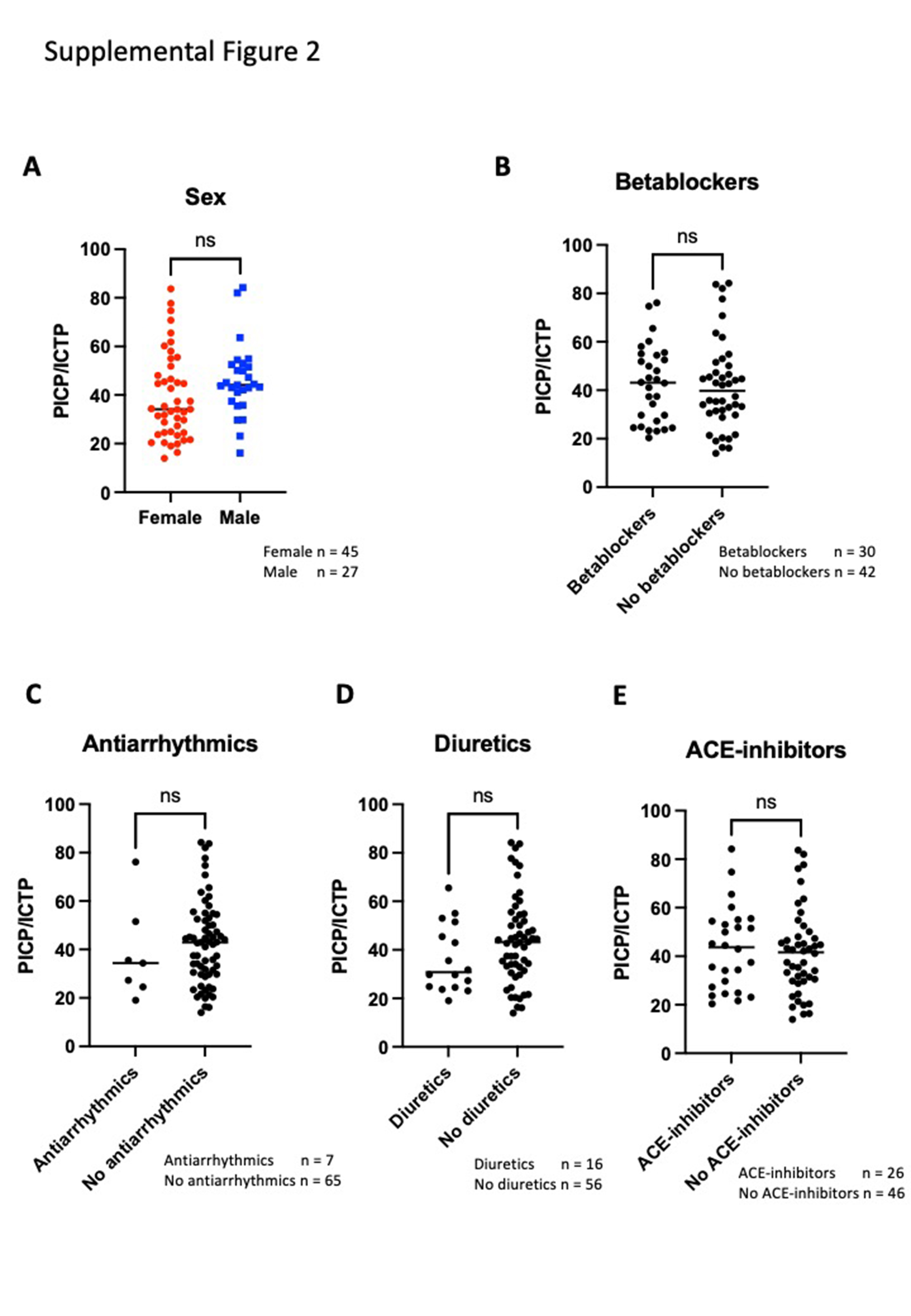

Supplement: Supplementary file 3 [file Image_2.jpg]
